# Hereditary Angioedema Therapy: Kallikrein Inhibition and Bradykinin Receptor Antagonism

**DOI:** 10.1097/1939-4551-3-S3-S34

**Published:** 2010-09-15

**Authors:** Marc Riedl

**Affiliations:** 1 Section of Clinical Immunology and Allergy, UCLA-David Geffen School of Medicine, Los Angeles, CA

**Keywords:** HAE, contact system, ecallantide, icatibant

## Abstract

Current strategies for the treatment of hereditary angioedema (HAE) include targeted inhibition or antagonism of the contact system, which is dysregulated in HAE patients by a C1 esterase inhibitor deficiency. Ecallantide, a plasma kallikrein inhibitor, and icatibant, a selective bradykinin-2 receptor antagonist, have recently been evaluated in clinical studies for the treatment of acute HAE attacks. Both drugs have demonstrated evidence of efficacy and safety in treating acute HAE episodes, with ecallantide approved for use in the United States and icatibant approved for use in Europe. As therapeutic options for HAE expand for both for prophylactic and acute treatment strategies, a number of patient-specific and drug-specific factors have emerged as important considerations when developing individualized HAE management plans. Optimization of HAE therapy will require further integration of new therapies into the current treatment paradigm.

## 

Hereditary angioedema (HAE) is a rare genetic condition caused by C1 esterase inhibitor (C1INH) deficiency and marked by episodic cutaneous, intestinal, or laryngeal swelling. HAE symptom frequency and severity is highly variable, but the unpredictable attacks of angioedema are frequently disabling and occasionally fatal. Historically, treatment options for HAE have been extremely limited in many parts of the world. Therapeutic agents recently developed for the acute treatment of HAE attacks can be categorized into 2 general groups: protein replacement therapies and medications targeted at specific single components of the contact pathway (Figure 1). C1INH replacement therapy is discussed elsewhere in the supplement. Reviewed here are 2 HAE therapies, ecallantide and icatibant, that target specific elements of the contact pathway.

**Figure 1:**
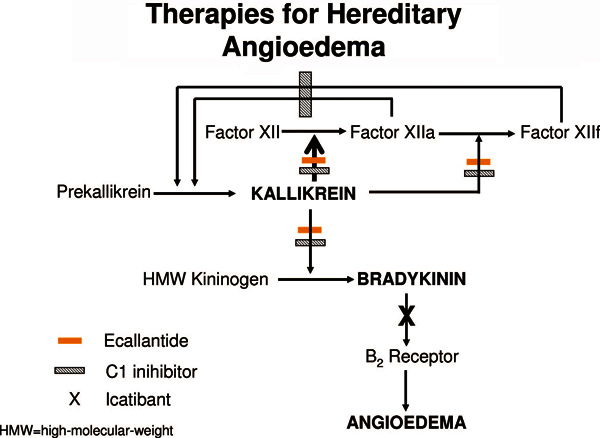
**Therapy for HAE is directed at controlling the dysregulated activity of kallikrein and bradykinin that is responsible for the clinical symptoms of HAE**. The hatched bar represents the inhibitory activity of C1INH, the orange bar the inhibitor activity of ecallantide, and the black × the receptor antagonism of icatibant. Source: Kaplan AP, Joseph K. The bra-dykinin-forming cascade and its role in hereditary angioedema. Ann Allergy Asthma Immunol. 2010;104:193-204.

Tissue angioedema episodes associated with C1INH deficiency are mediated by dysregulation of the contact system, ultimately leading to overproduction of bradykinin[1]. C1INH has numerous inhibitory functions within the contact pathway and within the complement and fibrinolytic systems[2]. Within the contact or kinin system, which is most relevant to the pathophysiology of the clinical angioedema of HAE, the most important inhibitory effects of C1INH are on Factor XIIa and kallikrein. In the absence of sufficient C1INH, factor XIIa effectively initiates a cascade leading to the local tissue production of bradykinin[3]. Factor XIIa accomplishes this by converting prekallikrein to kallikrein and also by activating a highly efficient autoactivation cycle, whereby factor XIIa acts on factor XII to produce additional factor XIIa[4]. This cycle efficiently up-regulates kallikrein production. Because C1INH also acts as a major inhibitor of kallikrein activity, inadequate C1INH concentrations permit unregulated kallikrein cleavage of high-molecular-weight kininogen (HMWK) to produce bradykinin. Data from numerous in vitro, animal, and human studies strongly support bradykinin as the major mediator of tissue angioedema in individuals with HAE[5][6][7].

The critical reactions of the contact pathway leading to HAE angioedema symptoms (factor XIIa → kallikrein → bradykinin) occur locally at the surface of endothelial cells[8]. The endothelial cell membrane appears to be a naturally occurring location for bradykinin production because Factor XII binds to a complex of the urokinase plasminogen activator receptor and cytokeratin 1 expressed on endothelial cell surfaces[9]. Circulating HMWK-kallikrein complexes preferentially bind to the receptor for the globular heads of C1q and cytokeratin 1 on the endothelial cell surface[10]. Localization of these components facilitates production of bradykinin, which then interacts with the bradykinin-2 receptor (B2R), also located on the endothelial cell surface[11]. Activation of B2R results in increased vascular permeability; release of associated nitric oxide and prostaglandin E, which augment vasodilatation; and resulting extravasation of fluid into subcutaneous tissue spaces (ie, angioedema)[6][12]. Thus, while C1INH acts at multiple sites to regulate the contact cascade, kallikrein and bradykinin are the critically dysregulated components leading to the clinical symptomatology of HAE. As a result, recent clinical investigations have included considerable focus on targeted therapies that 1) specifically inhibit kallikrein activity, thereby down-regulating bradykinin production, or 2) block bradykinin-mediated vascular effects, thereby preventing the endothelial permeability that ultimately leads to tissue swelling.

## Kallikrein Inhibition

Ecallantide (Kalbitor, Dyax, Cambridge, MA) is a 60amino-acid protein that was identified by phage-display technology, a process that allows large libraries of proteins to be screened and selected for specific functions or binding activity. Ecallantide (also known as DX-88), selected for its high affinity and specificity for human plasma kallikrein, inhibits kallikrein activity and thereby prevents bradykinin synthesis. Ecallantide is produced in a *Pichia pastoris *expression system, and though administered intravenously in early clinical development, the 30 mg dose used in Phase III studies is currently formulated for subcutaneous administration via 3 individual 1-mL injections. The plasma half-life of ecallantide is ~2 hours[13].

After preclinical and early clinical development, 2 Phase III clinical studies were performed to investigate ecallantide therapy for the treatment of acute HAE episodes. The 2 studies, termed EDEMA3 and EDEMA4 (*E*valuation of *D*X-88 *E*ffects in *M*itigating *A*ngioedema), were conducted as randomized, double-blind, placebo-controlled trials of treatment for cutaneous, abdominal, and laryngeal angioedema attacks in subjects with Type I or Type II HAE. The EDEMA studies used 2 unique patient-reported outcome measures with each having been specifically designed and validated by the study sponsor for the evaluation of HAE symptoms[14]. The Treatment Outcome Score (TOS) is a three-component tool that includes evaluation of anatomic sites affected, symptom severity, and change in symptoms over time. The TOS reflects overall improvement or worsening relative to baseline based on these patient-reported variables, with a possible range of 100 (significant improvement) to -100 (significant worsening), where 0 represents no change. Thus, an increased TOS indicates clinical improvement. The Mean Symptom Complex Score (MSCS) is a similar though somewhat less complex patient-reported outcome measurement. The MSCS is based on 2 factors: site(s) of swelling and symptom severity. The MSCS score range is 0 (no symptoms) to 3 (severe symptoms), so that lower MSCS represents clinical improvement. These validated measurement tools were useful and effective for regulatory purposes, though not particularly intuitive to clinicians or patients reviewing the study data.

EDEMA3 included 72 randomized subjects experiencing acute HAE attacks and had a primary end point of symptom improvement as measured by TOS at 4 hours after drug administration. Data from 69 subjects could be analyzed for this time point and showed a significant improvement for the ecallantide-treated group compared with placebo (mean TOS 63 vs 36, *P *= 0.045). At 24 hours after the dose, this treatment effect was maintained, with a statistically significant improvement for ecallantide over placebo (*P *= 0.02)[15].

**Figure 2:**
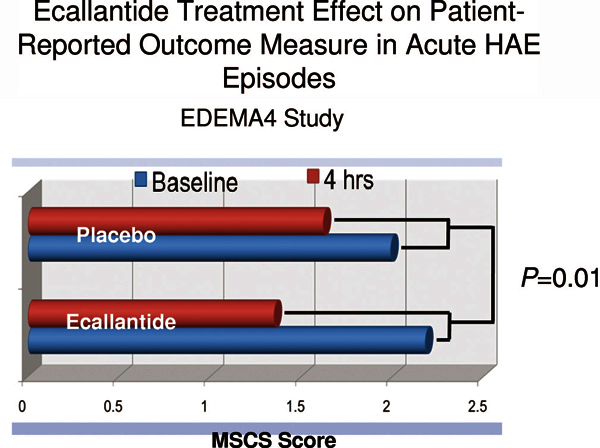
**The randomized, double-blind, placebo-controlled EDEMA4 study demonstrated a statistically significant improvement in the Mean Symptom Complex Score (MSCS) at 4 hours after treatment with ecallantide compared with placebo for acute HAE episodes**. Source: Developed from data provided in Table 7 of US Food and Drug Administration (www.fda.gov/ohrms/dockets/AC/09/briefing/20094413b1-03-Dyax.pdf).

EDEMA4 followed a study design very similar to EDEMA3, although the primary outcome was shifted to the change in patient-reported MSCS at 4 hours after drug administration. Ninety-six subjects with acute HAE symptoms were randomized to ecallantide or placebo, with results again demonstrating superior treatment outcomes for ecallantide compared with placebo. At 4 hours, 89 subjects had evaluable data, which showed a mean change in MSCS of -0.8 for ecallantide versus -0.4 for placebo (*P *= 0.01; Figure 2) This treatment effect was again evident at 24 hours after the dose with a greater mean reduction in MSCS for ecallantide versus placebo (- 1.5 vs - 1.1, *P *= 0.04)[15]. Thus, these 2 similar Phase III studies, which included 143 unique HAE patients, showed consistent results demonstrating the efficacy of ecallantide for acute HAE attacks.

With regard to safety, ecallantide was generally well-tolerated, with most reported side effects of mild severity and occurring with similar frequency in both drug and placebo groups. These included symptoms commonly seen with HAE attacks (abdominal pain, nausea) but also upper respiratory tract infections, headache, and fatigue[15]. However, throughout the course of the ecallantide clinical development program, 10 of 255 subjects developed systemic hypersensitivity reactions consistent with anaphylaxis. All reactions occurred with 60 minutes of dosing and all patients recovered completely without sequelae after appropriate medical treatment[16]. To date, the cause of these reactions is unclear, as no specific patterns or predictive risk factors have been identified. Anti-ecallantide antibodies have been detected in 12.9% of study subjects, including anti-ecallantide IgE in 2.1%; however, anti-drug antibodies do not seem to be strongly associated with hypersensitivity reactions[15][16]. Further, some subjects experiencing anaphylaxis were cautiously rechallenged after skin testing and tolerated ecallantide without systemic reaction. In contrast, one patient clearly had a reproducible acute anaphylactic reaction with a rechallenge procedure[16].

On the basis of the efficacy and safety data from EDEMA3 and EDEMA4, ecallantide was approved by the FDA in December 2009 for the treatment of acute attacks of HAE. The concern for hypersensitivity reactions prompted a boxed warning highlighting the potential risk of anaphylaxis and the need for the drug to be administered by a health care professional. Ecallantide is commercially available in the United States with a required Phase IV safety study instituted to track and investigate any additional occurrences of dose-related anaphylaxis.

## Bradykinin Receptor Antagonism

Given the role of bradykinin as the principal mediator of vascular permeability and tissue swelling in HAE, targeted blockade of bradykinin effects is a rational strategy for treatment. Icatibant (Firazyr, Shire HGT, Basingstoke, UK) is a second-generation BR2 receptor antagonist that has been investigated for the treatment of acute HAE attacks. Icatibant is a synthetic decapeptide with a structure similar to bradykinin but containing 5 nonproteinogenic amino acids. Thereby, it functions as a highly potent, selective, competitive antagonist at the B2R site and is not degraded by major bradykinin-metabolizing enzymes, so that it is more stable than bradykinin. Icatibant is formulated for subcutaneous administration as a single 3-mL (30-mg) injection and has a terminal half-life of 1 to 2 hours[17].

Two Phase III trials investigating the efficacy and safety of icatibant in treating acute cutaneous, abdominal, and laryngeal HAE attacks have been completed. The studies, known as FAST 1 and FAST 2 (*F*or *A*ngioedema *S*ubcutaneous *T*reatment 1 and 2) were both randomized, double-blind, controlled trials with nearly identical study designs, objectives, and endpoints. However, the FAST 1 study (JE409 #2103), conducted in North America, Argentina, and Australia, was placebo-controlled, whereas the FAST 2 study (JE409 #2102), conducted in Europe and Israel, used tranexamic acid treatment as the control/comparator. The primary endpoint for both Phase III studies was median time to onset of relief as determined by patient-reported visual analogue scales (VAS). The standardized VAS tool is a 100-mm scale ranging from 0 "no symptoms" to 100 "worst possible symptoms." Onset of symptom relief was defined as an absolute reduction from predose VAS of ≥ 20 mm for predose scores of 30-50 or ≥ 30 for predose scores >50. Initial onset of relief was determined retrospectively after the subject reported 3 consecutive time points with symptom relief[18].

FAST 1 included 64 treated subjects, with 56 subjects randomized and 8 subjects treated with open-label icatibant for laryngeal edema. Median time to onset of symptom relief was 2.5 hours for icatibant compared with 4.6 hours for placebo (*P *= 0.142). This difference in the primary end point was not statistically significant, although a number of secondary endpoints strongly supported significant improvement in the icatibant group compared with placebo. These included median time to regression (start of improvement) of symptoms (0.8 vs 16.9 hours, *P *< 0.001 favoring icatibant) and median time to overall patient improvement by physician assessment (1.0 vs 5.7 hours, *P *< 0.001 favoring icatibant)[18].

FAST 2 enrolled 77 subjects with 74 randomized and 3 subjects treated for laryngeal edema with open-label drug. Consistent with the effect observed in FAST 1, median time to onset of symptom of relief was 2.0 hours for icatibant but 12 hours for the tranexamic acid comparator arm (*P *< 0.001). Statistically significant results were observed for secondary endpoints as well, supporting the superior efficacy of icatibant compared with tranexamic acid for the treatment of acute HAE attacks[18].

No serious adverse events or systemic hypersensitivity reactions were identified in the clinical studies of icatibant. Reported adverse drug effects were generally mild, with the most common being local symptoms at the subcutaneous injection site. Injection site reactions were reported in most patients receiving icatibant and included symptoms of erythema, burning, pruritis, and swelling. Such reactions were self-limited, lasting 10 minutes to a few hours, and did not seem to be associated with any risk of more serious reactions. No subjects withdrew from the studies because of these local reactions. Icatibant does not seem to be immunogenic, although no reliable antibody test exists[18].

**Figure 3:**
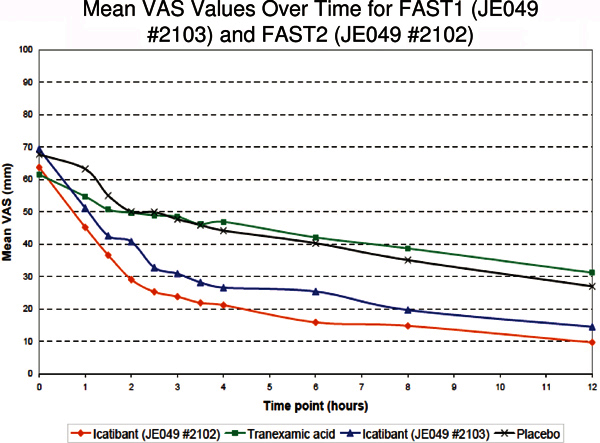
**Mean posttreatment VAS scores over time for icatibant and comparator treatment of acute HAE episodes in the randomized, double-blind FAST 1 and FAST 2 studies**. Source: European Medicines Agency (http://www.ema.europa.eu/ humandocs/PDFs/EPAR/firazyr/H-899-en6.pdf).

Because of the primary endpoint outcome in FAST 1, icatibant failed to obtain FDA approval for use in the United States. Factors contributing to the FAST 1 study outcome are not entirely clear, although a surprisingly robust placebo effect was evident. Additionally, it has been proposed that the analytical approach (responder analysis) likely contributed to the lack of statistical significance. An alternative analysis, examining change from baseline at the 4-and 12-hour time points, did demonstrate the statistical superiority of icatibant at both timepoints[18]. On the basis of the clinical efficacy and safety data of FAST 1 and FAST 2 (Figure 3), icatibant was approved for the treatment of acute HAE by the European Medicines Agency in July 2008 and is currently prescribed in several European countries and Brazil. An additional Phase III study of icatibant for acute treatment of HAE was initiated in 2009 with the goal of obtaining sufficient data to obtain FDA approval.

## Optimizing Hereditary Angioedema Treatment

The recent development of multiple therapies for the treatment of HAE has increased the availability of effective medications. In some instances, individuals with HAE have unprecedented therapeutic options. These advances provide opportunities to optimize the medical care and quality of life for HAE patients, but are accompanied by both practical and societal challenges. Providers treating HAE will need to consider a number of important factors when consulting with and managing the treatment of individual patients.

Because of the wide variability in symptoms for individual HAE patients, treatment strategies will ideally take into account a number of patient-specific factors[19]. Clearly, these include the frequency and severity of angioedema symptoms, which may principally determine whether regular prophylactic therapy or intermittent on-demand acute therapy is most beneficial. Rapidity of attack progression and access to acute medical care may also play a role when considering long-term prophylactic versus as-needed therapy. The frequency of variability in the therapeutic response to new agents and their adverse effects have not yet been fully determined. Variability is evident in individual responses to prophylactic C1INH therapy,[20] and adverse effects are well demonstrated by the rare hypersensitivity reactions to ecallantide[21]. Research efforts to better define or predict this variability will improve the tailoring of therapy to individual patients.

Drug-specific features will also be important in therapeutic decision-making. Though study protocol differences make direct comparisons of data difficult, the C1INH products, ecallantide and icatibant seem to have comparable treatment effects,[15,18,22,23] so that clinical efficacy does not seem to be a strong factor for distinguishing among them. With regard to safety, plasma C1INH products have a long and extensive history of safe use despite a theoretical risk of transmission of infectious agents. Some concern exists for allergic reactions to recombinant C1INH and ecallantide, but recent study data demonstrate this to be a greater concern for ecallantide, with its small but real risk of hypersensitivity reactions[15,24]. Icatibant appears to have an excellent safety profile to date, although clinical experience is somewhat limited. Route of drug administration is currently a distinguishing feature. C1INH products are presently approved only for intravenous use, which may present logistical challenges in some situations. There is considerable interest in the use of C1INH products subcutaneously; however, at present this remains in clinical development. Consequently, because of the unpredictability of HAE attacks, patients may face challenges in rapidly accessing IV products through clinics or emergency departments. Self-infusion programs and infrastructure are likely to improve this situation, though not every patient will be comfortable with this approach. Subcutaneous products may be attractive for many patients, but the risk of hypersensitivity reactions with ecallantide likely precludes home self-administration at present. Finally, medication costs will be a factor for most HAE patients and health care organizations, representing a difficult issue that each region or country must address. Drug development for rare conditions is an expensive endeavor, and while medication policies and pricing vary nationally, health care expenses for serious, chronic medical conditions are an important societal issue in most communities. Thus, with new therapies comes the challenge of devising individualized management plans for each patient that will reduce the morbidity and disability of HAE, minimize treatment complications, and remain sustainable for long-term management.

In summary, major advances in therapy for HAE have occurred in recent years, with an increase in effective treatment options for this rare and often devastating condition. Though patients and providers are still determining how to optimally incorporate newer medications into HAE management plans, a number of important treatment goals may eventually be realized. With proper training, home treatment of angioedema attacks may be possible with self-infused C1INH products or eventually with subcutaneous icatibant or ecallantide. Based on previous studies, such self-treatment is expected to reduce the duration of attacks compared with hospital treatment and to minimize the detrimental impact on patient lives[25,26]. Long-term prophylactic C1INH therapy and effective available acute therapy may ultimately reduce requirements for attenuated androgens in some patients, thereby reducing the toxicities and complications occasionally associated with androgens[27]. Most importantly, these therapies provide reliable life-saving and life-changing relief from the unpredictable attacks suffered by HAE patients around the world.
